# Dynamically Scalable NoC Architecture for Implementing Run-Time Reconfigurable Applications

**DOI:** 10.3390/mi14101913

**Published:** 2023-10-07

**Authors:** Qaiser Ijaz, Hiliwi Leake Kidane, El-Bay Bourennane, Gilberto Ochoa-Ruiz

**Affiliations:** 1 ImViA Laboratory, University of Burgundy, 21000 Dijon, France; hiliwi-leake.kidane@u-bourgogne.fr (H.L.K.); ebourenn@u-bourgogne.fr (E.-B.B.); 2 Department of Computer Systems Engineering, Islamia University of Bahawalpur, Bahawalpur 63100, Pakistan; 3 School of Engineering and Science, Tecnologico de Monterrey, Zapopan 45201, Mexico; gilberto.ochoa@tec.mx

**Keywords:** FPGA, reconfigurable computing, scalable NoC, underutilized IPs, partial reconfiguration, dynamic partial reconfiguration

## Abstract

The paper proposes two architectures for a dynamically scalable network-on-chip (NoC) for dynamically reconfigurable intellectual properties (IPs) to save power. The first architecture is a run-time scalable column-based NoC, where the columns of the NoC are scaled up and down at run-time depending on the demands to connect reconfigurable IPs. The second architecture is an extension of the first, where both the rows and columns of the NoC are dynamically scaled up and down on demand. A robust control manager is developed to control the IP and sub-NoC reconfigurations by optimizing the reconfiguration costs. The proposed architectures have been implemented and tested in actual prototypes on a Virtex 6 FPGA mounted on the ML605 board. The results show that dynamically scalable architectures are capable of significant power reduction as compared to traditional static architectures for the same size of the NoC. It is anticipated that the scalable NoC can be very useful for sharing the FPGA resources among IPs at runtime.

## 1. Introduction

The sustained use of a Field Programmable Gate Array (FPGA) as a shared resource requires multi-tenancy. It refers to the ability to accommodate multiple use cases in a cloud-like environment or serve multiple applications as a shared hardware accelerator. In such scenarios, it is not efficient to map all the flexible use cases as a static design into the FPGA fabric. Dynamic and Partial Reconfiguration (DPR) enhances processing flexibility in FPGAs, where configuration can be altered during operation to meet user or environmental needs. This capability presents several opportunities for energy conservation. Firstly, the utilization of dedicated hardware enables the establishment of ideal implementations in relation to processing capabilities and energy efficiency. By using DPR in a wide range of situations, it is possible to improve resource utilization, which leads to smaller reconfigurable units and lower static power consumption. Dynamic reconfiguration facilitates the alteration of clock configuration, specifically enabling dynamic frequency adaptability and performance variation, in order to regulate power consumption and enhance energy efficiency as needed. Furthermore, it can be employed to deactivate the routing of clock signals to specific FPGA resources. This enables the implementation of a clock gating strategy with minimal impact, yielding noteworthy outcomes. However, reconfiguration and hardware implementation costs are complex and must be understood to determine if gains outweigh costs.

The utilization times of different intellectual property (IP) cores vary within an FPGA system. Using the DPR to reduce the communication resources used by the underutilized IPs brings communication constraints. The communication requirements of each IP are not always the same, and when one IP is replaced by another, the communication medium needs to adapt to the requirements of the new IP. This established the need for an optimal and adaptive communication medium. A dynamically scalable network-on-chip (NoC) capable of adapting to the dynamic needs of the system.

The design of dynamically scalable NoCs is the subject of a substantial amount of research that includes adapting routing algorithms and switching techniques, as well as changing buffer sizes and topologies. Adapt-NoC [1] allowed for the flexible configuration of multiple sub-NoCs, each of which can be tailored to the specific needs of the applications currently in use. With the intention of balancing load and reducing network latency, researchers in [2] developed a novel regional routing algorithm based on area partitions. An addition of a router was proposed in [3], the added router is removed when the IP leaves the system. The drawback of this technique, however, is that it gives less attention to the complexity introduced due to the increased number of reconfigurable regions for each router and the power consumption per single router configuration. Researchers in [4,5,6,7] have proposed run-time reconfigurable topologies to introduce dynamic flexibility to communication needs. Different adaptive routing algorithms have been proposed in [8,9,10,11,12,13,14] for dynamically reconfigurable NoCs that are capable of avoiding deadlock and obstacles by bypassing faulty components. The functional examples [15] of FPGA partial reconfiguration and the accompanying design tools are in abundance. Likewise, researchers have proposed different routers [16,17,18] with dynamic behavior. The authors in [19,20,21] have all proposed virtualized FPGA-based hardware accelerators for intensive applications. Reconfigurable regions of the FPGA are virtualized to be shared among different dynamically reconfigurable accelerators. A reconfigurable packet-switched NoC [22] is where switches are added and removed to adapt dynamically, and a global control unit is used to update the routing table and maintain continuity of the routing while new switches and modules are inserted. Most of the related works share a general topic, but reconfigurability is proposed only for specific infrastructures and protocols. Two surveys [23,24] summarized and classified the related works with respect to topology, routers, links, routing, and switching techniques. The authors have highlighted the important aspects, like the building block, concept, objective, size, toolchain and, most importantly, the cost.

Even though adding and removing a single router has its own disadvantages in terms of reconfiguration cost, topology irregularity, and routing complexity, there is a tradeoff between the power needed for reconfiguration and the power saved after replacing the router with bypass links. The power requirement to bypass links is significantly lower than the power requirement for routers. However, when the reconfiguration frequency is high, such an approach might be counterproductive. In addition, adding or removing single routers can introduce unexpected complexity and irregularities in the topology. In view of these shortcomings, improved, dynamically scalable architectures are designed and evaluated for reduced power consumption. The proposed architectures, instead of dynamically adding or removing the system’s possibly underutilized IPs, dynamically change the number of the NoC’s routers. The proposal’s novelty is that the reconfiguration is performed in groups of routers instead of fine-grained reconfiguration, with the goal of minimizing the cost of partial reconfiguration. The system starts with a set of static (always-on) IPs, and then the network expands or shrinks based on IP demand by adding or removing columns of routers for the column architecture or both rows and columns for the row/column architecture. In the case of router reclaiming, direct links substitute the routers in order to maintain connectivity with the remote routers and reduce power consumption. The primary contributions are the two architectures that can be scaled up or down dynamically. However, the architecture has a complete set of essential components that are instrumental in delivering the promised performance. This includes user-defined IPs that can be reconfigured, an adaptive XYX routing method that can handle changes in the topology, and a strong control manager that manages all the IPs, checks the status of the routers, and reconfigures the IPs and NoC.

Section 2 presents the two architectures of a dynamically scalable NoC and other essential components of the system. Section 3 addresses reproducibility by laying out the detailed experimental setup. Section 4 presents the experiment scenarios and results. Section 5 discusses the real-world application of the architectures under discussion.

## 2. Architecture

In general, not all IPs in a given system are required all the time. As a result, keeping the basic IPs used for the system to run as static, the rest can be designed as dynamically reconfigurable IPs to share the resources of the FPGA in a time-multiplexing manner. To do so, it is necessary to analyze which IPs will be designed as static and which IPs need to be designed as dynamically reconfigurable. The IPs frequently used in the system can be grouped as static and the other underutilized IPs as reconfigurable. Then, this IP grouping will be used as a guide to define the architecture and size of the dynamically scalable NoC.

**Figure 1:**
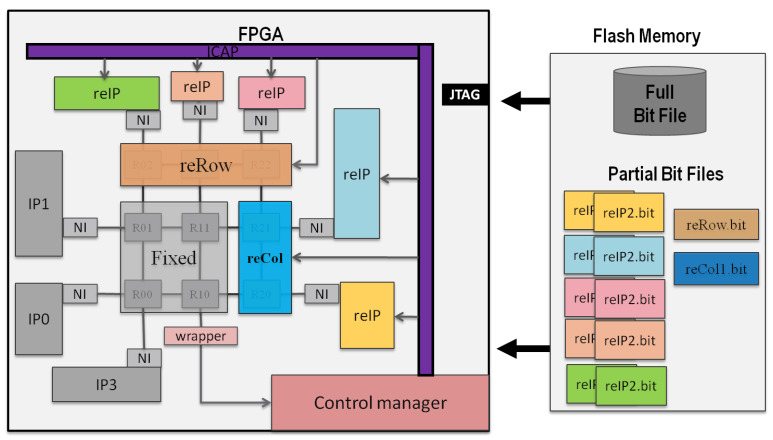
Dynamically scalable NoC-based reconfigurable IPs.

In the proposed architecture, as shown in Figure 1, there are three static IPs in gray color and five reconfigurable regions, marked as reIP, to be shared by dynamically reconfigurable IPs. The reconfigurable bitstreams of the IPs are stored on the right side of the figure in the flash memory, but they can also be stored in DDR memory for fast reconfiguration.

The reconfigurable regions defined for the reIPs are interconnected via the network interface (NI) of the NoC. However, in many cases, not all the reconfigurable regions defined for IPs will be required. Therefore, some reconfigurable regions and the routers connected to them might be idle. As a result, it is more convenient to remove the idle routers and replace them with bypass links until they are required by the system to reduce power consumption.

The NoC is split up into a set number of fixed sub-NoCs and reconfigurable rows (reRows) and columns (reCols) of routers. The routers in these rows and columns can be both added or subtracted according to the need for the IPs, dynamically at runtime. This reconfigurable topology enables the growth and degrowth of routers using DPR. The static part of the NoC or the routers in the static part of the network do not have this functionality. So, when the NoC is in reconfiguration mode, only the routers surrounding the sub-NoC (the static part) to be reconfigured will be affected. The rest of the NoC will forward packets. To avoid packets currently in the reconfiguration routers being lost, the control manager will inform the router in the reconfiguration sub-NoC to clear their buffers and not accept new packets.

The full and partial bitstreams required to configure the FPGA are generated and stored in non-volatile flash memory. The IP and sub-NoC reconfiguration is managed by a microprocessor-based control manager, which is also incorporated into the design. The control manager monitors the status of the reconfigurable regions, reconfigurable IPs, and routers.

Initial system steps include powering on the static sub-NoC and any associated base IPs. The remaining IPs are dynamically reconfigured at runtime per the control manager’s instructions. Then, depending on the demand for connections to any of the reconfigurable IPs, the NoC will be scaled up by adding rows and columns of routers. Similarly, when IPs are no longer required (i.e., following task completion), the manager scales down the NoC by removing the idle reconfigurable sub-NoC.

In the current era, there are plenty of demands for different application scenarios from end users. Similarly, the demand for FPGA-based hardware accelerators is increasing over time. However, it is impossible to design all applications and hardware accelerators as static architectures within a single FPGA. In order to manage access to FPGA resources and reduce power, DPR has been considered an optimal solution to share the FPGA resources among IPs at runtime.

However, as the reconfiguration process incurs non-negligent time and power, the IPs need to be analyzed and studied. Their activation frequency has to be correctly assessed, and the resources they need have to be determined, which influences the decision on which reconfigurable regions can be floorplanted. This information is gathered at the design phase and can help to design the more frequently used IPs as static and the rest as dynamically reconfigurable modules.

But, even after designing dynamically reconfigurable IPs, not all of the reconfigurable regions will be active 100% of the time. This implies that when the IP connected to any reconfigurable region is idle, the router connected to it will also be idle. This rationale leads, therefore, to the fact that power consumption can be optimized by deactivating idle routers.

Multiple copies of bitstreams corresponding to different reconfigurable regions are generated for each IP, introducing flexibility in the placement of IPs. When only one IP is running in a reconfigurable row or column of routers, the IP can be replaced in another reconfigurable region.

The IPs are designed in a way to send a one-packet message to the control manager to inform them of their task completion. To facilitate this, a small kernel is added to each IP to send a message to the control manager. The packet contains three flits, where the first flit is the destination address of the control manager, the second flit corresponds to the physical address of the reconfigurable region, and the third flit is the logical address of the IP.

### 2.1. Dynamically Scalable NoC

A dynamically scalable NoC mainly focuses on scaling up and down the number of switches in the network. The rationale for the proposed architecture is to save power by activating only the routers connected to active IPs at runtime. Two approaches or architectures of dynamically scalable NoC are proposed: in the first approach, only the columns are designed as scalable, and in the second approach, both columns and rows are designed as scalable.

**Figure 2:**
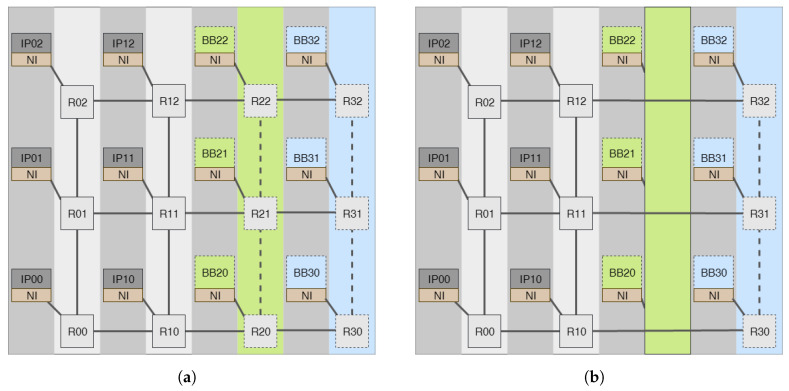
Column-wise scalable 4 × 4 NoC with dynamic behavior. (**a**) State of all routers used. (**b**) State of links replacing the routers in green column.

The goal of this design is to preserve a regular 2D mesh even after groups of routers in the same column have been added or removed. As a result, the NoC’s row size will remain constant while the column size adapts on the go. Direct connections are made between the routers in each column and the columns to the right and left of that column while the column of routers is idle, as shown in Figure 2. For example, the routers in the third column of Figure 2a are replaced by direct links in Figure 2b. This strategy helps to reduce power consumption and reduce latency as the number of intermediate hops decreases. Since the electronic components of the direct links are very small compared to the components of the router, this approach clearly reduces power consumption. Similarly, replacing the idle intermediate router with a direct link will reduce the latency by the clock cycle required to traverse one router.

### 2.2. Adaptive XYX Routing

**Figure 3:**
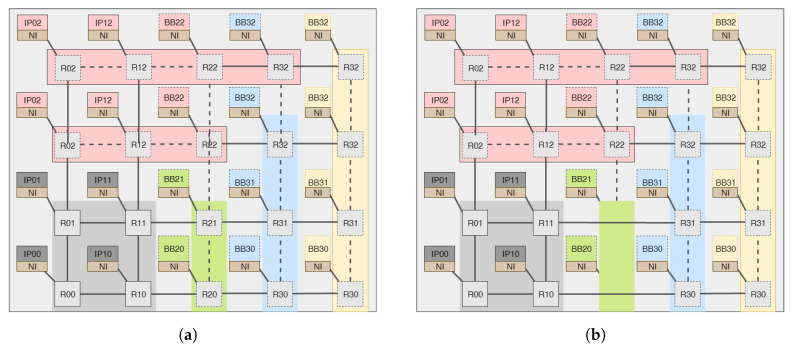
Row/column-wise scalable 4 × 5 NoC with dynamic behavior. (**a**) State of all routers used. (**b**) State of links replacing the routers in green column.

Figure 3 illustrates how our proposed architecture dynamically scales both the NoC’s columns and rows. The NoC’s 4 × 5 mesh architecture is broken up into 2 × 2 static sub-NoCs and a number of sub-row and sub-column components that may be rearranged on the run. For instance, if the frequently used IPs are only the 4 IPs connected to the static sub-NOC, then the system will activate only the static sub-NoC at boot and dynamically scale up when there is a request for a new router until it reaches its maximum capacity in Figure 3a. Then, when some IPs connected leave the network after task completion, the NoC will scale down by removing any of the reconfigurable idle sub-NoCs. For example, if the IPs connected to routers R21 and R20 have finished their task and left the system, then the routers will be replaced by a direct link marked by a green area as shown in Figure 3b. As it is observed from the figure, the topology’s regularity is somehow distorted. In this case, a special fault-tolerant routing method is required so that the NoC can deal with such dynamic gaps. We have proposed an adaptive new routing method to deal with such complexity.

The complexity of Figure 3 is in the routing algorithm. Normally, when the NoC is in full scale or there are no inner sub-NoCs replaced by direct links, the XY routing algorithm can be used. In the XY routing, the packet header flit carries the destination node address. Then, the intermediate routers compare their routing addresses with the destination router addresses. Depending on the comparison result, the routers select the appropriate output port for the packet. The routing algorithm first checks for the X-coordinate (east–west) alignment and, if the packet’s current x-coordinate address is aligned with the destination address x-coordinate, then the packet flit header moves in the south or north direction until it arrives at the destination router.

**Figure 4:**
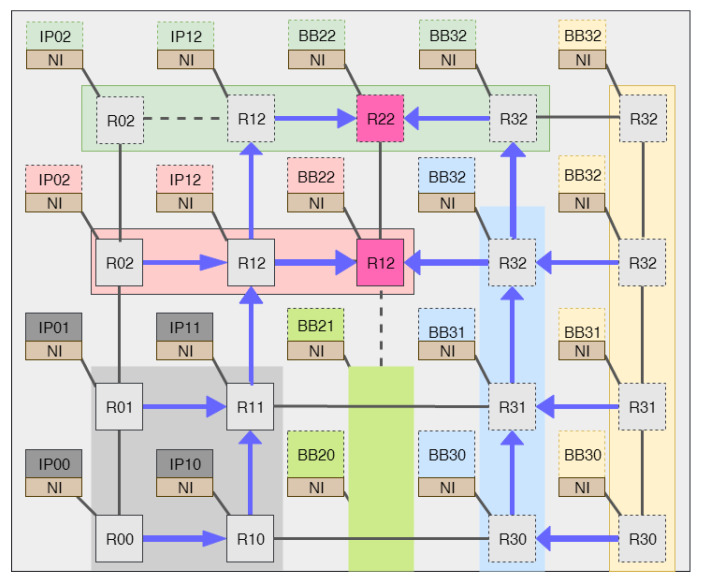
Adaptive XYX routing when sub-column replaced by direct links.

When a direct link replaces intermediate routers, it is not possible to route packets to their destinations using the conventional XY routing algorithm. For example, if router R00 in Figure 4 wants to send a packet to router R22 using XY routing, first router R00 will forward the packet to router R10 and then router R10 will compare the destination x-index value with its x-coordinate and, for sure, the destination index will be greater than the current address, and the router R10 will choose the east port to forward the packet to router R30. In router R30, the destination x-index value will be less than the value of R30 x-coordinate, and the router will be forced to choose the west port. This will result in a deadlock.

The solution that we have proposed is to use an adaptive XYX routing, as illustrated in Algorithm 1. The control manager will dynamically assign the routers with reconfigurable topology to any of the two modes, and they will support both XY and XYX routing. Let us verify if the packets sent from router R00 in Figure 4 could reach the destination address BB22 connected to router R23, assuming that the XYX routing mode of the adjacent routers to the sub-column marked in green is activated. First, router R00 will choose the east port and forward the packet to router R10. Since the XYX routing mode of R10 is activated, which indicates the adjacent router is replaced by a direct link, it will check the value of the y-index of the destination address and forward the packet to the north port. In a similar manner, Router R11 will choose the north port, and when the packet arrives at Router R12, it will be forwarded to its destination to reach its x-index destination address.

**Figure 5:**
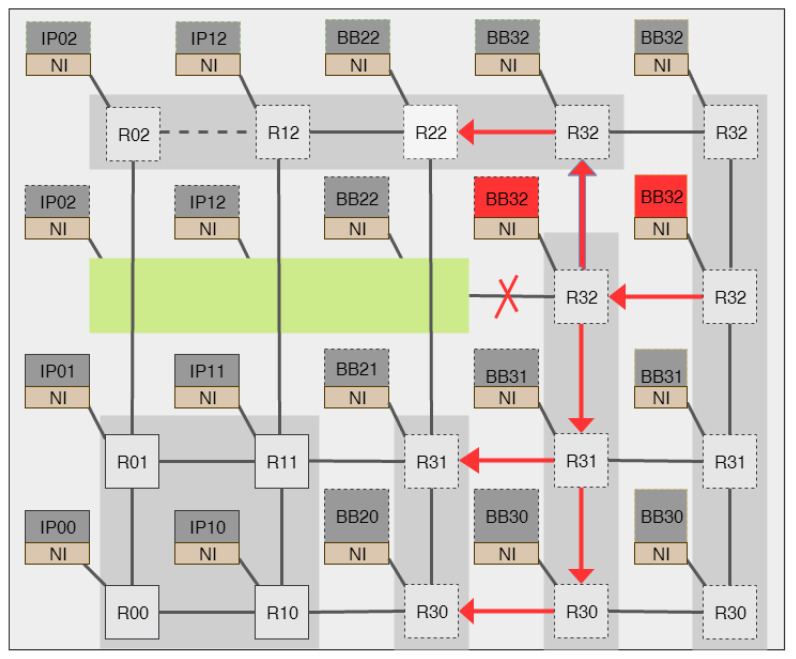
Adaptive XYX routing when sub-row replaced by direct links.

It will be the same case when a sub-row is replaced by a direct link, as shown in Figure 5 in the green area. If either router R32 or R42 wants to send packets to routers below or above the green region using the traditional XY routing algorithm, the packets will stack at the output west port of router R32. The solution is to use the XYX routing algorithm. For example, if the IP connected to router R42 wants to send packets to the IP connected to router R01, the packets have to follow W(x)−>S(y)−>W(x)−>W(x)−>W(x) to arrive at the destination.
**Algorithm 1** XYX Routing  1:**procedure** xyx routing  2:    Xi:address of surrounding router in X direction  3:    Yi:address of surrounding router in Y direction  4:    Xr:position of removed row in X direction  5:    Yr:position of removed column in Y direction  6:    Xd:destination address in X direction  7:    Yd:destination address in Y direction  8:    *For each router (Xi, Yi) neighbors to removed columns or rows*:  9:    **if** Xr=Xd **then** //destination in the same Row10:        follow Yi.11:        **close**;12:    **else if** Xr<XdorXr>Xd **then** //destination in different Row13:        then follow Xi.14:        **close**;15:    **else if** Yr=Yd **then** //destination in the same Column16:        then follow Xi.17:        **close**;18:    **else if** Yr<YdorYr>Yd **then** //destination in different column19:        then follow Yi.20:        **close**;21:    **end if**22:**end procedure**

To solve this problem, router routing algorithms must be dynamically adapted to XYX routing. The surrounding routers must modify their routing algorithms when a direct link dynamically replaces a column or a row. Since the packet will not forward in a backward direction, livelock will not happen. To avoid the deadlock possibility, the control manager updates the routing table prior to reconfiguration.

### 2.3. Configuration Control Manager

Dynamic sub-NoC reconfiguration, application-requested new-module placement, and router topology changes are all handled by the control manager. Since the idea of DPR is to share the resources of the FPGA in a time-multiplexing manner, it is a must to make sure that any IPs to be mapped onto a given reconfigurable region will not run at the same time. Consequently, the number of reconfigurable regions is defined considering the maximum number of possible IPs that will run concurrently.

In order to facilitate IP placement and cater to a flexible placement of IPs, a bitstream of each IP, corresponding to different reconfigurable regions, is generated. This strategy enables a more flexible placement of the IPs at runtime. The control manager stores all the data pertaining to the potential placements and uses it to select the best placement at runtime. The bitstreams of the reconfigurable IPs and run-time reconfigurable sub-NoCs are generated at design time. Since the static IPs and the static sub-NoC are designed with the initial static configuration, they are mapped first when the system is initialized. Once running, the control manager handles any new application requests.

During the reconfiguration of a new task, the control manager has to optimize the power cost due to frequently reconfigured IPs and the gain after replacing the IPs with blank bitstreams. Normally, when the reconfiguration frequency increases over a given duration of time, the power cost incurred due to reconfiguration increases as well. As a result, the control manager has to set a counter for the reconfiguration for each IP and count how many times they have reconfigured within a given time. Then, if an IP has reconfigured more frequently, the control manager decides to keep it running in the reconfigurable region without reconfiguring. However, in order not to affect the dynamic scaling of the sub-NoCs, the control manager will place this frequently used IP near the static sub-NoC.

**Figure 6:**
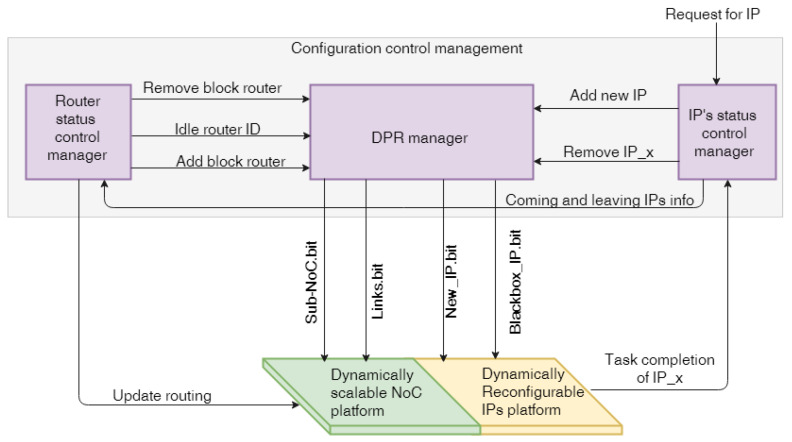
Block diagram of the configuration control manager.

The control manager is encompassed by the three blocks, as shown in Figure 6. The first block is the IP’s status control manager responsible for IP status control, and the second block refers to the router’s status control manager. The third block is responsible for handling the partial reconfiguration process by loading the correct bitstream into the corresponding reconfigurable region.

The IP-status Control Manager (IP-CM) is responsible for managing the status of running IPs. The IPs are designed to send one packet message, indicating the completion of their task. To do so, the IP’s status control manager communicates with the router in the static part via a wrapper. When an IP is removed from the network after completion, the NI immediately sends the packet to the IP-CM. Then, the IP’s status control manager has to inform the RS-CM to include the specific router in its list of idle routers. Similarly, the reconfiguration manager is informed to replace the reconfigurable module, either by mapping a new IP or by sending a blank bitstream.

The IP-CM is also in charge of handling requests for new IP addresses. The status control manager of an IP requests that the reconfiguration manager load the requested IP if the IP wants to connect with an IP that is not currently running and mapped to the FPGA. In addition to these activities, the IP status control manager must set a counter for each IP to measure the frequency of reconfiguration. Then, it will list the often reconfigured IPs and notify the reconfiguration manager that they should be kept static.

The Router Status Control Manager (RS-CM) is primarily responsible for tracking the status of all of the routers in the system, even those that are now idle. Then, it asks the reconfiguration manager to add or delete groups of routers so that IPs may communicate effectively while using as few of the system’s resources as possible. In other words, directly bypass links replace a column or row of idle routers to save energy, since the power required for the router’s bitstreams is greater than that of the direct links. As will be seen in the adaptive routing section below, it is also necessary to dynamically adjust the routing mode to accommodate the new setup.

The DPR Manager (DPR-M) requires information about incoming and removed IPs, as well as the status of any idle routers and, finally, any requests to add and remove blocks of sub-NoCs. Moreover, it is also in charge of evaluating the availability of a given reconfigurable region and checking whether a bitstream can be loaded or not in the PRR before performing the dynamic reconfiguration.

## 3. Methods

The proposed run-time scalable NoC architectures were implemented, starting with the Hermes NoC [25]. First, we generated different sizes of NoCs based on mesh topologies using the Atlas NoC generation tool [26]. Then, we modified the HDL architecture of the NoC to split it into reconfigurable and static parts, based on the requirements of the proposed architecture. The network interfaces (NI) connecting the NoC components and the IPs are designed to be static. User-defined IPs are defined to characterize the dynamically scalable NoC. These user-defined IPs perform simple computations. The three fixed IPs are designed to manipulate addition, subtraction, multiplication, and division sequentially. The reconfigurable user-defined IP accepts integers and commands from the user through the control manager. The proposed architectures were implemented targeting the Virtex-6 FPGA (XC6VLX240T) [27] mounted in the ML605 board.

We used Xilinx ChipScope Pro [28] for power measurements. The tool inserts several analyzers right into your design to observe internal signals and nodes, including integrated hard or soft processors. The analysis of power gains in the context of DPR is challenging, due to the intricate nature of the interconnections involved. Accurately quantifying the energy loss is crucial, as the reconfiguration process inherently introduces additional overheads. The best practice is to use power models derived for DPR, and we chose the coarse-grained DPR model, one of the three power models, as proposed in [29]. The three proposed DPR models help tailor estimation accuracy to analysis and detail levels. A fine-grained model is always preferred for precision, but its high elaboration complexity may make it unsuitable in practice. When reconfiguration overhead is reduced compared to hardware power, fine power specifics become secondary. Hence, the coarse-grained model is suitable for estimating power in various scenarios, including ours.

The source code for the dynamically scalable NoC architectures is available in a GitHub repository. The link is available under the Data Availability Statement.

## 4. Results

**Figure 7:**
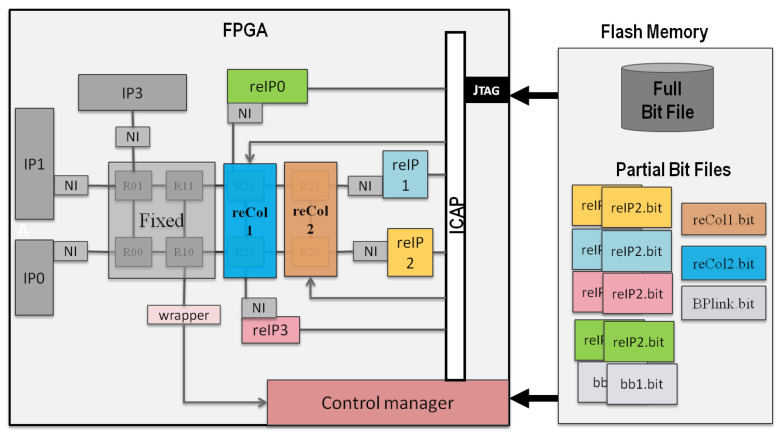
The case of a 2 × 4 scalable NoC.

Figure 7 presents a NoC of size 2 × 4, divided into two columns of 2 × 2 static sub-NoCs and two columns of 1 × 2 dynamically reconfigurable sub-NoCs marked as reCol1 and reCol2. The physical address is designed to be global, to reduce the complexity between the static and dynamic parts of the network, enabled by reconfigurable topology.

Three fixed and eight dynamically reconfigurable IPs are used in the experiment. The control manager’s wrapper and the three static IP addresses make up the static portion. When the FPGA is loaded with the full bitstream, only the static sub-NoC and static IPs are activated. Then, the reconfigurable sub-NoC columns are activated on demand. Out of the eight reconfigurable IPs, four are underutilized and are placed in the reconfigurable regions connected to reCol2. This will help to deactivate the farthest of the columns of reconfigurable columns, as the IPs reconfigured at the far end will be used only 10% of the time.

Table 1 compares the performance of run-time scalable NoCs against that of a static full NoC linked to the same dynamically reconfigurable IPs. When the idle column of the NoC is replaced by direct links or blank bitstreams, the findings reveal a considerable reduction in power consumption, 17.6849%. There is a significant power decrease when we compare the entire static NoC power consumption presented in the first line of the table to the dynamically scalable column-based NoC presented in the second line of the table.

The resource utilization of the reconfigurable sub-NoC columns, their bitstream size and the reconfiguration time required to load into the FPGA, are presented in Table 2. From the result, it is seen that the bitstream size of most of the reconfigurable sub-NoCs is small. The reconfiguration time is also close to 280 microseconds.

Recall Figure 1 of a 3 × 3 full-scale NoC, divided into a 2 × 2 static sub-NoC, one 1 × 2 scalable column sub-NoC, and one 3 × 1 scalable row sub-NoC. There are thirteen IPs used in this experiment. Three IPs are static and connected to the static sub-NoC. The remaining 10 IPs will share the five reconfigurable regions marked as reIP, connected to the dynamically reconfigurable column and router via the static NI. The six reconfigurable IPs connected to the reconfigurable row of sub-NOCs are underutilized, and they will be used only 10% of the time. The four IPs connected to the reCol are moderately utilized, being active 30% of the time.

The power consumption using the dynamically scalable row/column-based NoC is presented in Table 3. The first row presents the power consumption when a full static NoC of 3 × 3 is used for the same number of static and reconfigurable IPs. The aforementioned dynamically scalable NoC’s power usage is shown in the second row. Using the dynamically scalable row/column-based NoC reduced power usage by 16.2722%.

The resource utilization, bitstream size, and reconfiguration time needed to load the bitstreams of scalable row/column-based NoC are presented in Table 4. From the result, the bitstream size of most of the reconfigurable sub-NoCs is found to be small, and the reconfiguration time is also close to 280 microseconds, as they are proportionate to each other.

Since the performance of the proposed architecture is more related to how frequently the IPs are reconfigured, a comparison of the reconfigurable frequency vs. power is presented in Table 5. The IPs completed their tasks in less than 25 s. However, we have reconfigured them at different times and measure the power consumption cost. First, the IPs are reconfigured every 30 s, which is 10 times in a duration of 5 min. Then, they are reconfigured every minute and once every 5 min. The results in the table indicate that when a frequently utilized IP is designed as reconfigurable, the power cost increases due to repeated reconfiguration cost. Thus, it is necessary that the control manager has to analyze each reconfigurable IP, to check how frequently are reconfigured each IPs in a given time and the IPs which are reconfigured more frequently are required by the system and they need to stay there without reconfiguring.

Another important part that needs a compromise between reconfiguration and idle time is the reconfiguration of sub-NoC. The reconfiguration control manager records the reconfiguration frequency of the sub-NoCs, and if the idle time is less than five times the idle time of frequently reconfigurable IPs, then the reconfigurable sub-NoC will be marked as static. However, this threshold is not static, as the control manager needs to adapt to any new challenges or behaviors of the system over time.

Even though the scalable column-based NoC is not complicated like the scalable row/column-based, the first architecture has drawback in the latency as the scaling is one dimensional. A latency comparison of the two proposed scalable NoCs is presented in Table 6. For the experiment, a 4 × 4 row/column scalable NoC and 2 × 8 scalable columns-based NoC are considered. Both architectures have 16 routers, and both start with a 2 × 2 static sub-NoC. Then, the scalable column-based NoC grows only in one direction, the scalable row/column-based NoC grows in both directions.

Twenty packets of 16 flits length are forwarded and the latency measured. Compared to a scalable column-based NoC, the latency of a scalable row/column-based NoC is lower. This stems from the fact that when the NoC grows only in one direction, the maximum number of intermediate nodes increases, which results in higher latency.

## 5. Discussion

The real-world application of these architectures is in contemporary data centers, which are using FPGAs to enhance both the processing capacity and the energy efficiency of their facilities. The incorporation of hardware accelerators into cloud computing environments has been shown to enhance overall system performance and computational efficiency. Additionally, the utilization of FPGAs presents the opportunity for adaptive accelerators during runtime via the implementation of partial reconfiguration techniques. This enables the conservation of resources and power by allowing underutilized accelerators to share the same resource through time multiplexing. Similarly, using a NoC-based communication channel makes it easier for hardware accelerators, reconfigurable regions, the control manager, and the gateway to end users to talk to each other at the same time. We performed some foundational work by developing two service models [30]. With dynamically scalable architectures, those service models can be perfected and utilized in any academic or industrial-scale FPGA-based datacenter.

## 6. Conclusions

Two dynamically scalable architectures have been designed and evaluated for reduced power consumption. The proposed architectures, instead of dynamically adding or removing the system’s possibly underutilized IPs, dynamically change the number of the NoC’s routers. The novelty is that the reconfiguration is performed in groups of routers instead of fine-grained reconfiguration, with the goal of minimizing the cost of dynamic and partial reconfiguration. The system starts with a set of static (always-on) IPs, and then the network expands or shrinks based on IP demand by adding or removing columns of routers for the column architecture or both rows and columns for the row/column architecture. In the case of router reclaiming, direct links substitute the routers in order to maintain connectivity with the remote routers and reduce power consumption. The architecture has a complete set of essential components that are instrumental in delivering the promised performance. A robust control manager is developed to control the IP and sub-NoC reconfigurations by optimizing the reconfiguration costs. The proposed architectures have been implemented and tested in actual prototypes on a Virtex 6 FPGA mounted on the ML605 board. The results show that dynamically scalable architectures are capable of significant power reduction as compared to traditional static architectures of the same size. These architectures have the high usefulness of sharing FPGA resources with multiple users as a cloud service.

## Figures and Tables

**Table 1:** Power consumption comparison for the size 2 × 4.

	No of Static Routers Routers	No of Reconfig. Routers	No of Underutilized IPs	Power (mW)
2 × 4 Static NoC	8	0	4	311
2 × 4 Dynamically Scalable Column-based NoC	4	4	4	256

**Table 2:** Resources, bitstream size, and reconfiguration time of a 2 × 4 scalable column-based NoC.

DPR Module	Resources Utilization				Bitstream Size	Reconfig. Time
	LUT	FF	DSP	BRAM		
2 × 1 column	2013	4208	12	8	21 KB	0.28 ms
direct links	113	410	0	0	16 KB	0.23 ms

**Table 3:** Power consumption comparison for the size 3 × 3.

	No of Static Routers Routers	No of Reconfig. Routers	No of Underutilized IPs	Power (mW)
3 × 3 Static NoC	9	0	5	338
3 × 3 Dynamically Scalable Row Column-based NoC	4	5	5	283

**Table 4:** Resources, bitstream size and reconfiguration time of 2 × 4 scalable row/column-based NoC.

DPR Module	Resources Utilization				Bitstream Size	Reconfig. Time
	LUT	FF	DSP	BRAM		
2 × 1 column	2013	4208	12	8	21 KB	0.28 ms
direct links	113	430	0	0	16 KB	0.23 ms
1 × 3 row	3619	5121	40	12	36 KB	0.36 ms

**Table 5:** Reconfiguration frequency per 5 min vs. power cost.

Recon. Frequency per 5 min	Power Cost in mW
10	164
5	59
1	17

**Table 6:** Latency comparison of 4 × 4 and 2 × 8 NoC to deliver 20 packets of 16 flits length.

NoC Type	Num. Intermediate Router	Latency (Cycles)
4 × 4 row/column-based NoC	5	571
2 × 8 column-based NoC	7	1108

## Data Availability

The source code of the architectures is openly available in a publicly accessible GitHub repository at https://github.com/eQaiserIjaz/DSNoC under the GNU Affero General Public License v3.0.

## Abbreviations

The following abbreviations are used in this manuscript:

DPR	Dynamic and Partial Reconfiguration
DPR-M	Dynamic and Partial Reconfiguration Manager
FPGA	Field Programmable Gate Array
HDL	Hardware Description Language
IP	Intellectual Property
IP-CM	IP-status Control Manager
NoC	Network on Chip
PRR	Partial Reconfiguration Region
RS-CM	Router Status Control Manager
